# Supplementary material for: Timing variability and midfrontal ~4 Hz rhythms correlate with cognition in Parkinson’s disease
Source: NPJ Parkinsons Dis. 2021 Feb 15;7:14. doi: 10.1038/s41531-021-00158-x (PMC7884691; doi:10.1038/s41531-021-00158-x)
Supplement: Supplementary file 1 — Supplementary Information [file 41531_2021_158_MOESM1_ESM.pdf]

Interval timing variability and midfrontal ~4 Hz rhythms correlate with cognitive dysfunction in Parkinson's disease

Arun Singh[1], Rachel C. Cole[2], Arturo I. Espinoza[2], Aron Evans[3], Scarlett Cao[4], James F. Cavanagh[5] and Nandakumar S. Narayanan[2*]

Supplementary Figures

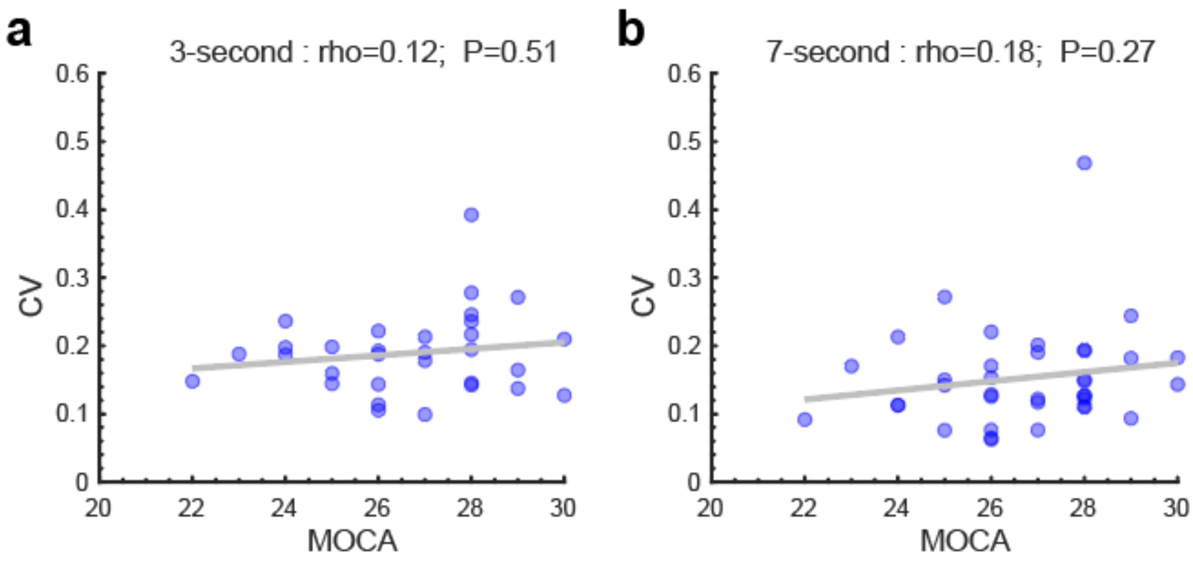

Supplementary Figure 1. Timing variability does not correlate with cognitive dysfunction in controls. Timing variability as measured by keypress CV correlated with cognitive function as measured by MOCA for a) 3-second and b) 7-second intervals in control participants only.

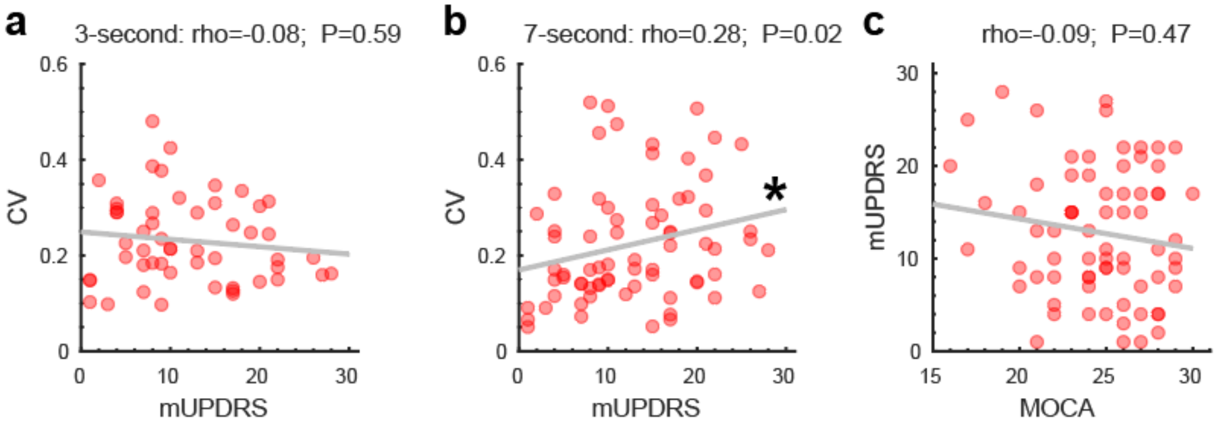

Supplementary Figure 2. Motor function by mUPDRS correlated with CV and MOCA in PD. Keypress CV correlated with motor function as measured by the mUPDRS for a) 3-second and b) 7-second intervals (*=p<0.05). c) mUPDRS was not correlated with cognitive function as measured by MOCA.

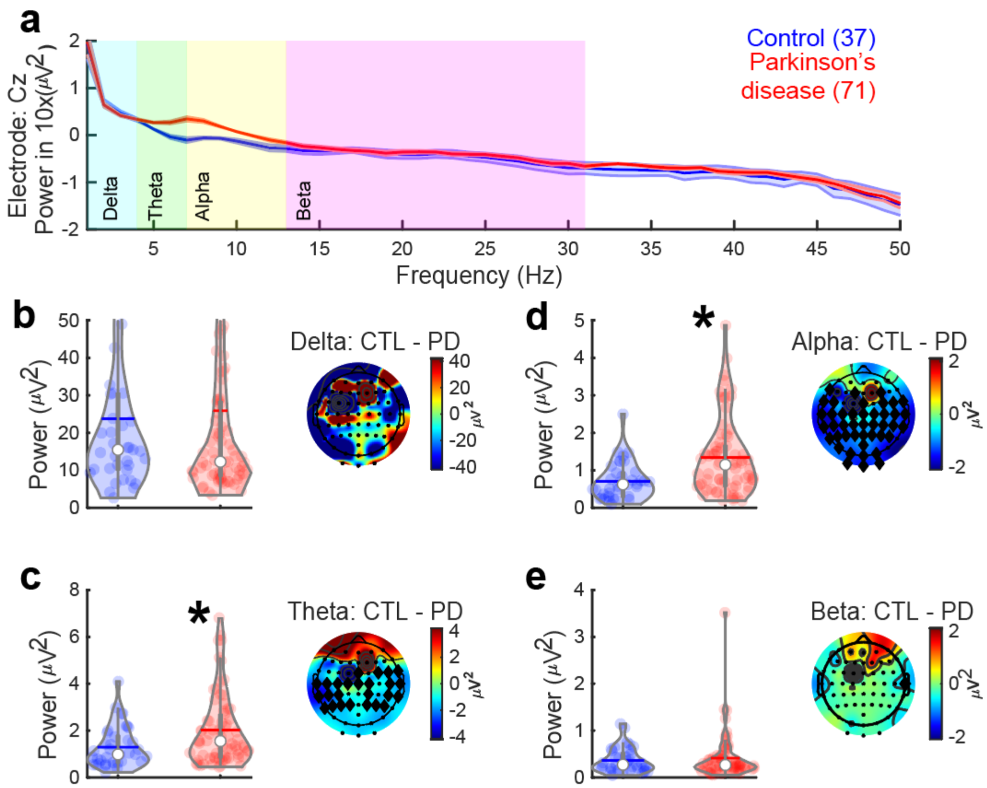

Supplementary Figure 3. Baseline midfrontal theta and alpha power is increased in PD. We also performed conventional spectral analysis on pre-stimulation data following Welch's power spectral density estimation method, using the pwelch function in MATLAB. We selected a 1-second time window prior to the imperative "Go" cue on 7-second trials. The number of overlapped samples was set to 50% of the window length and the non-equispaced fast Fourier transform (NFFT) was assigned as the length of the segments. a) Resting-state power spectral density of EEG power at midfrontal electrode Cz revealed marked increases in theta (4-7 Hz) and alpha (7-13 Hz) power for control (blue) and PD patients (red). Resting-state epoch was 1 second prior to the instructional text from 7-second interval trials. Power for control and PD patients for b) delta bands (1-4 Hz); c) theta bands (4-7 Hz; * = $t_{(106)}$=-2.70, p=0.008); d) alpha bands (7-13 Hz; * = alpha: $t_{(106)}$=-3.56, p=0.001); and e) beta bands (13-30 Hz).

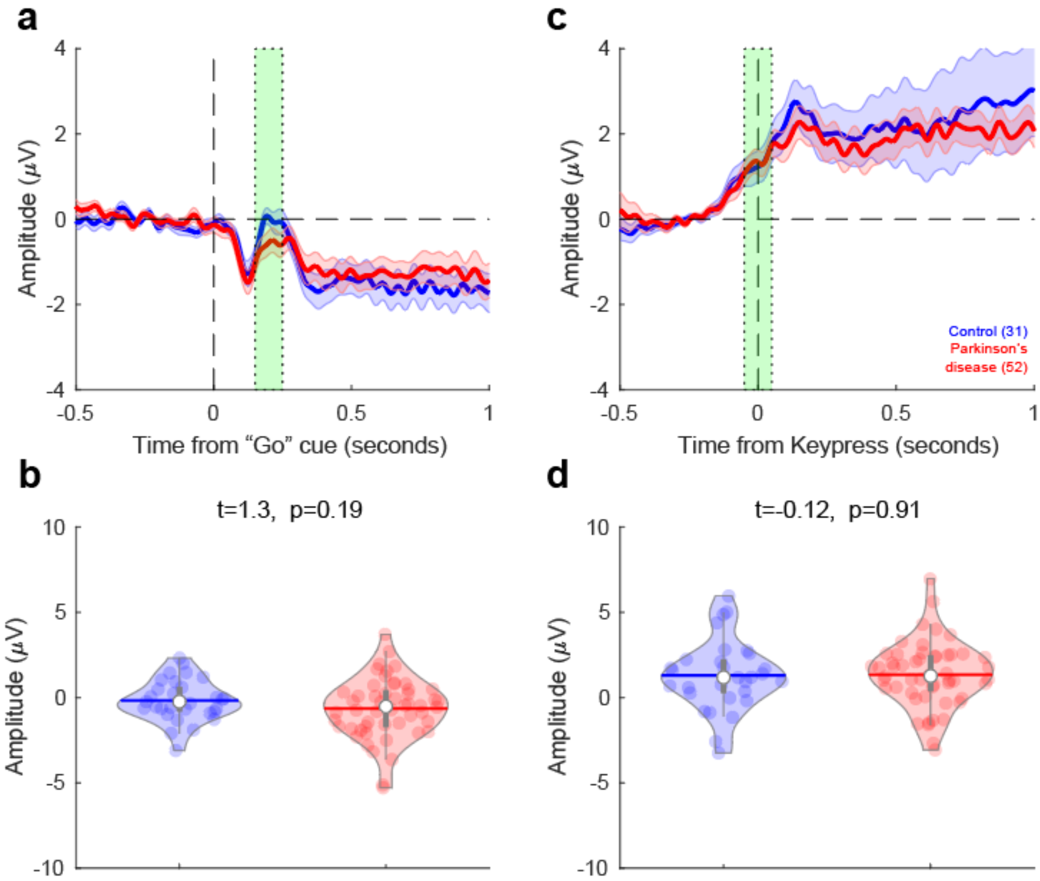

Supplementary Figure 4. Event-related potentials (ERP) from electrode Cz for 3-second trials. No reliable difference in midfrontal cue-triggered (a and b) and response-triggered (c and d) ERPs during 3-second interval timing task was observed between PD patients and control subjects (ROIs: green box).

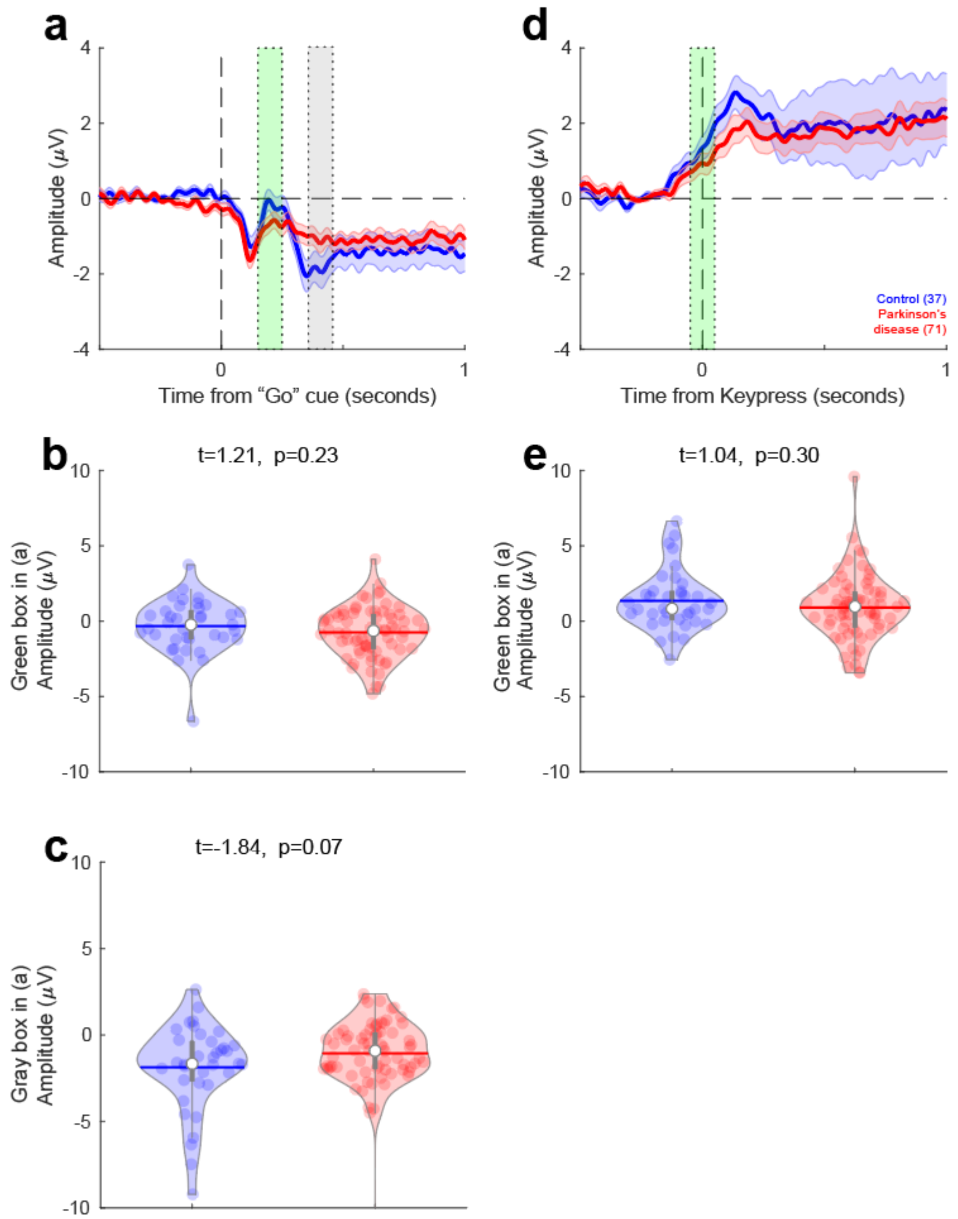

Supplementary Figure 5. Event-related potentials (ERP) from electrode Cz for 7-second trials. No reliable differences in midfrontal cue-triggered (a, b, c, and d-e) and keypress-triggered ERPs during 7-second interval timing task was observed between PD patients and control subjects (ROIs: green/gray box for cue-triggered analysis, green-box for keypress-triggered analysis).

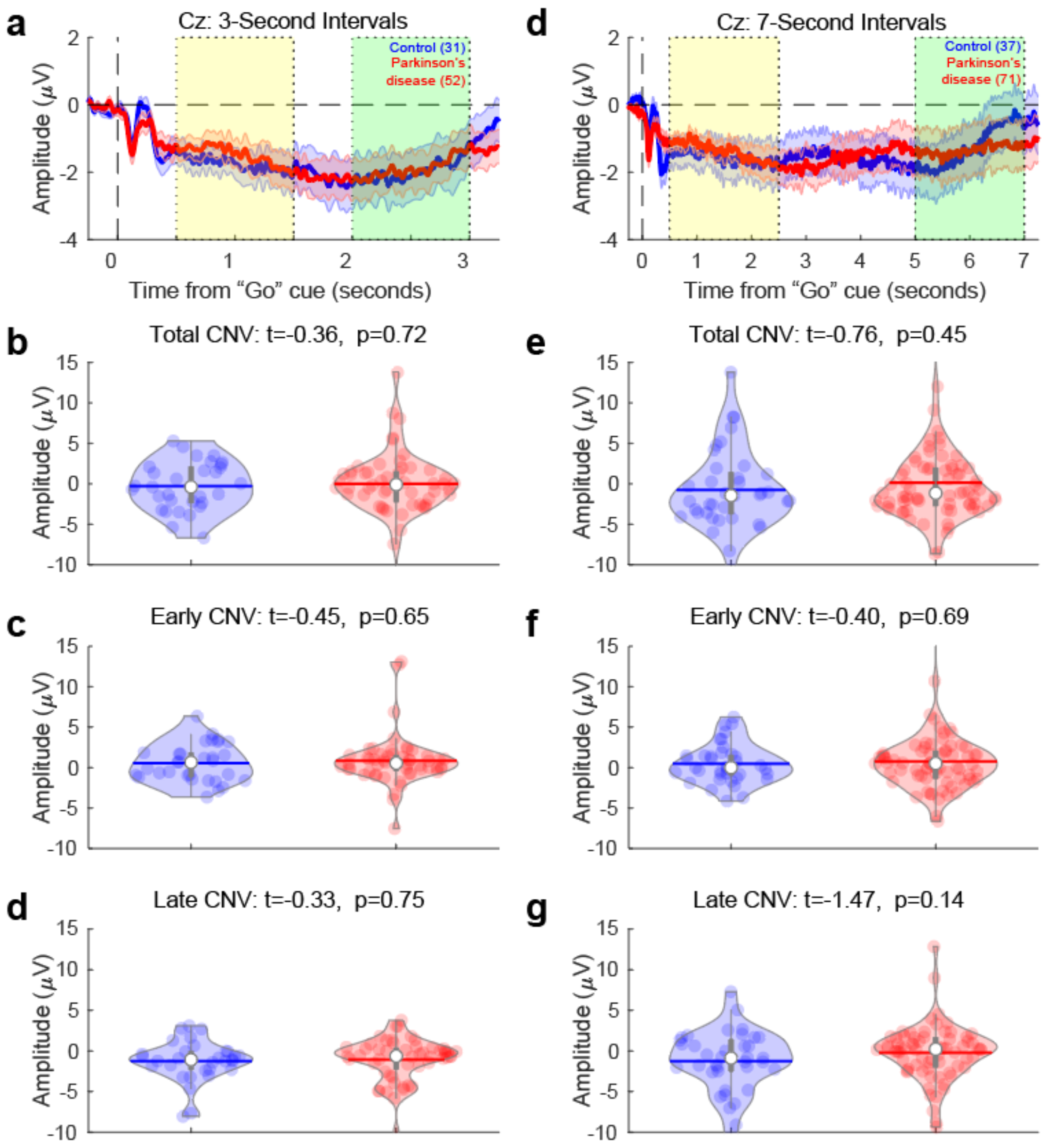

Supplementary Figure 6. Contingent-negative variation (CNV) analyses for early and late epochs. For 3-second intervals, a) we found no reliable differences in midfrontal CNV activity between control and PD participants for the mean amplitude of b) total CNV (0.5 - 3 seconds), c) early CNV (0.5 – 1.5 seconds), or d) late CNVs (2-3 seconds). We also observed no reliable differences for 7-second intervals for e) total CNV (0.5-7 seconds), f) early CNV (0.5-2.5 seconds), or g) late CNVs (5-7 seconds).

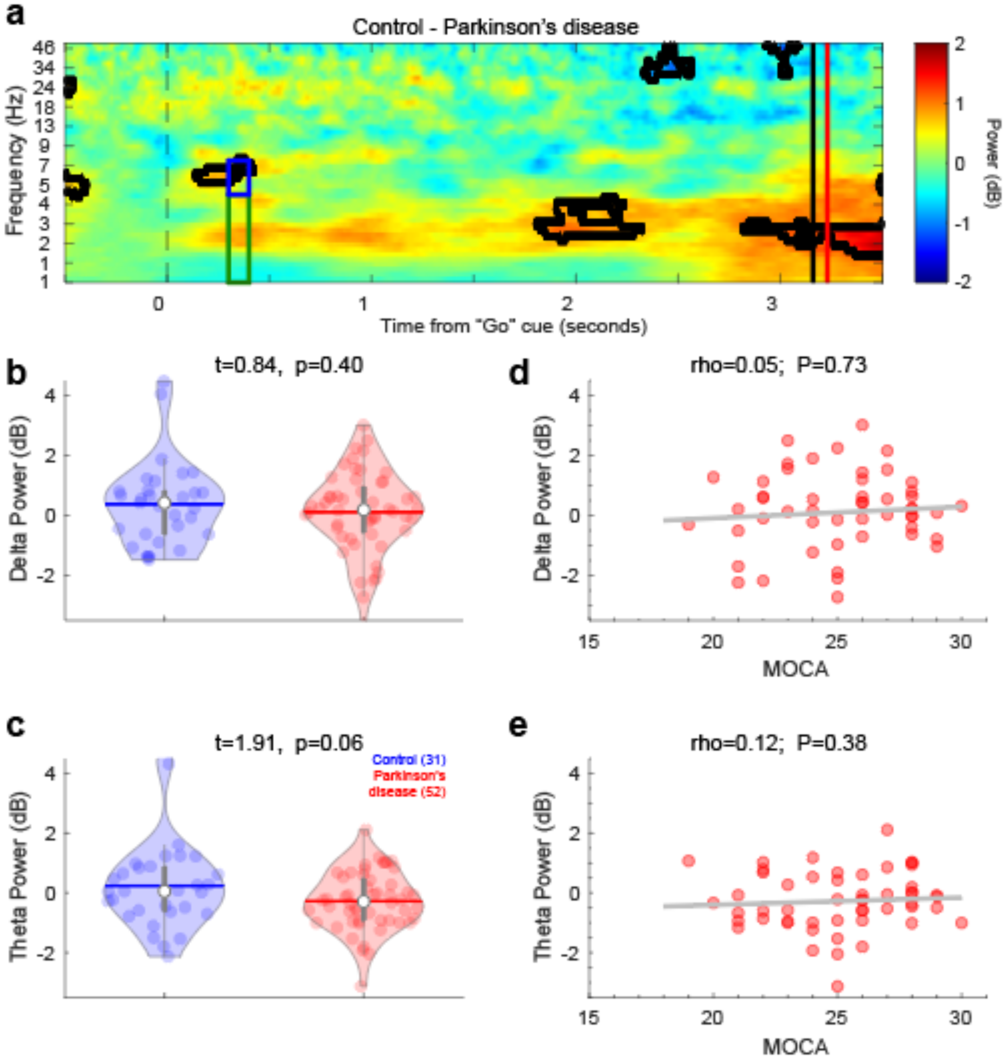

Supplementary Figure 7. Midfrontal delta/theta activity for 3-second intervals. a) We compared time-frequency power of midfrontal activity after the imperative “Go” cue for 3-second trials. Solid black lines indicate $p<0.05$ via a t-test of activity in control compared to PD patients. b) cue-triggered midfrontal delta power (1-4 Hz, tf-ROI: green box in a) and c) cue-triggered midfrontal theta power (4-7 Hz, tf-ROI: blue box in a) in PD patients. d) delta and e) theta power vs. cognitive dysfunction as measured by MOCA in PD patients. Data from PD (n=52) and control (n=31) patients on 3-second trials.

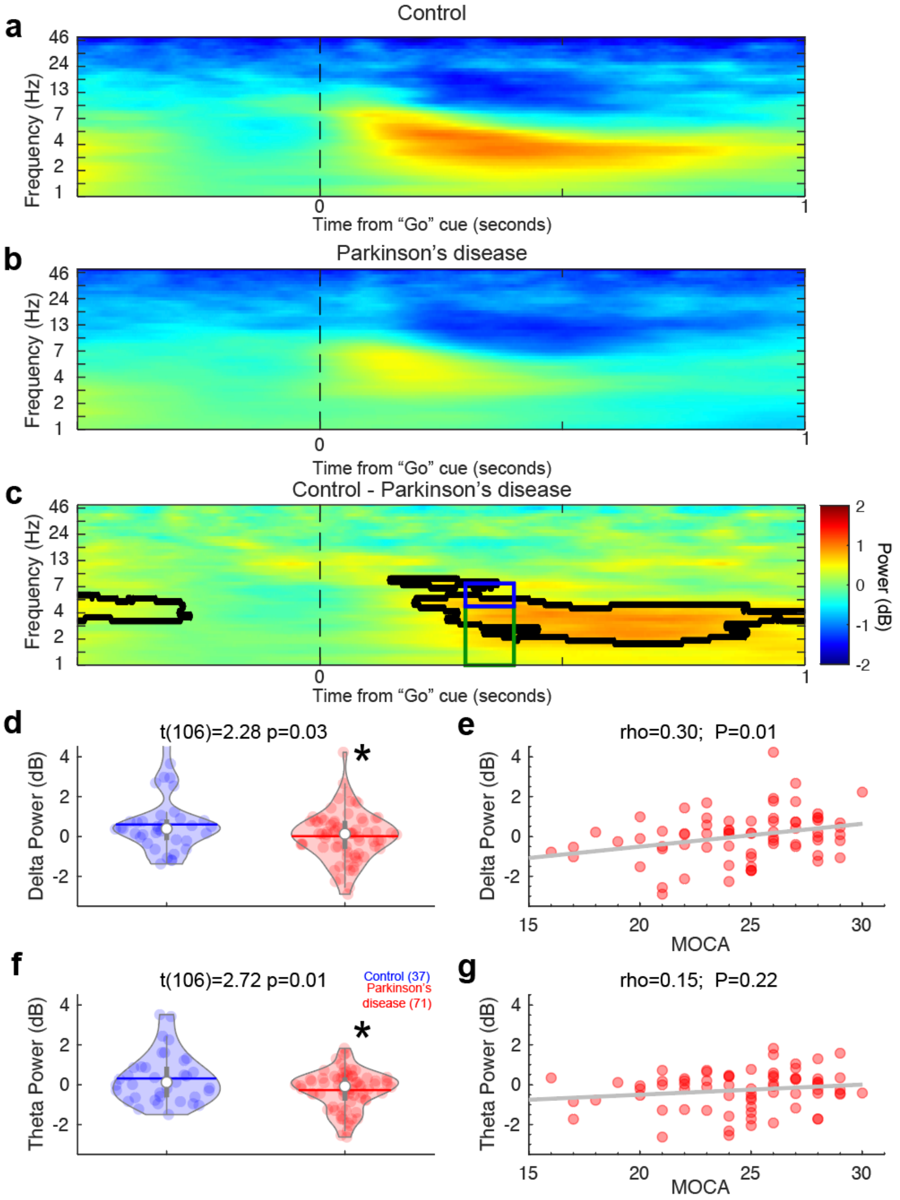

Supplementary Figure 8. Cue-triggered delta power from a cluster of midfrontal electrodes (FCz, Cz, and CPz) predicts cognitive dysfunction in PD. Whereas data in Figure 2 included electrode Cz according to our *a priori* hypothesis, data in this figure includes an expanded midfrontal coverage including FCz, Cz, CPz. Frequency power of midfrontal activity over time from the imperative "Go" cue from a) control and b) PD

participants on 7-second trials. c) Comparison of control and PD patients. Areas outlined by solid black lines indicate $p<0.05$ via a t-test of activity in control compared to PD participants. There was significantly less d) cue-triggered midfrontal delta power (1-4 Hz, time-frequency-Region-of-interest (tf-ROI): green box) and f) cue-triggered midfrontal theta power (4-7 Hz, tf-ROI: blue box) in controls vs. PD patients. e) Delta power predicted cognitive dysfunction as measured by MOCA in PD patients, but g) theta power did not. *=$p<0.05$. Data from control (n=37) and PD (n=71) patients.

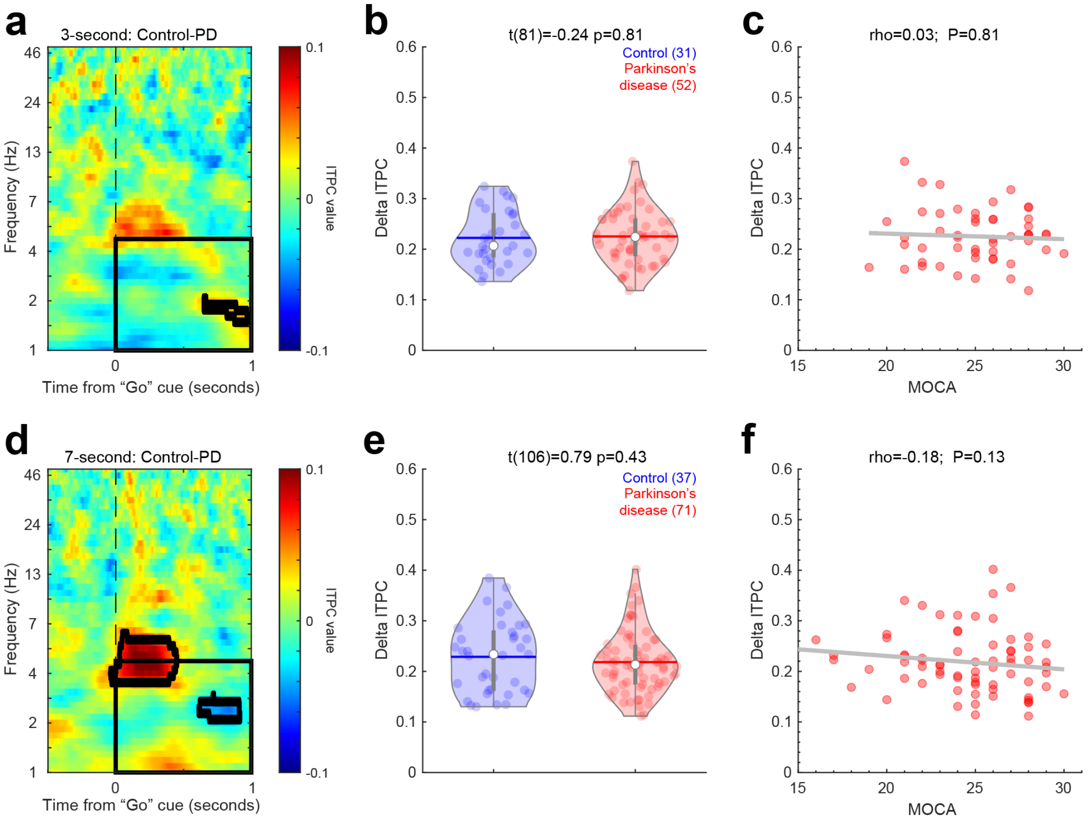

Supplementary Figure 9. Intertrial phase coherence (ITPC). a) For 3-second trials, there were, b) few overall consistent differences between Control and PD patients for delta bands 0-1 seconds after the cue, and c) no relationship with MOCA. Data from 24 ± 1.4 (mean ± SEM) trials. d) For 7-second trials, there were marked differences in ~4 Hz ITPC around the “Go” cue, but e) no overall differences in delta bands 0-1 seconds after the cue, and f) no relationship with MOCA. Data from 33 ± 0.8 trials.

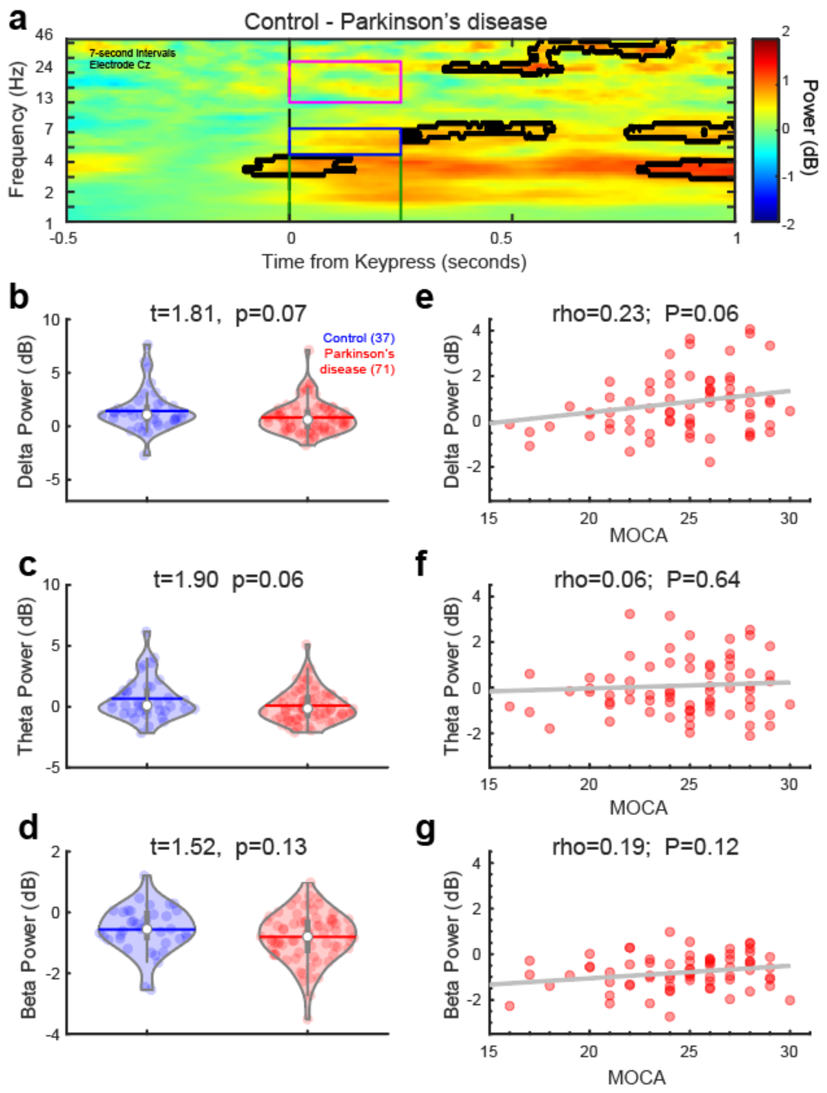

Supplementary Figure 10. Midfrontal activity around keypress. a) We compared time-frequency power of midfrontal activity around response ("keypress"; time=0). Areas outlined by solid black lines indicate p<0.05 via a t-test of activity in control compared to PD participants. b) delta power (1-4 Hz, tf-ROI: green box in a), c) theta power (4-7 Hz, tf-ROI: blue box in a) in PD patients, and d) beta power (13-30 Hz, tf-ROI: magenta box in a) in control vs. PD patients. e) delta, f) theta, and g) beta power do not significantly correlate with cognitive dysfunction as measured by MOCA in PD patients. Data from 71 PD and 37 control patients on 7-second trials.

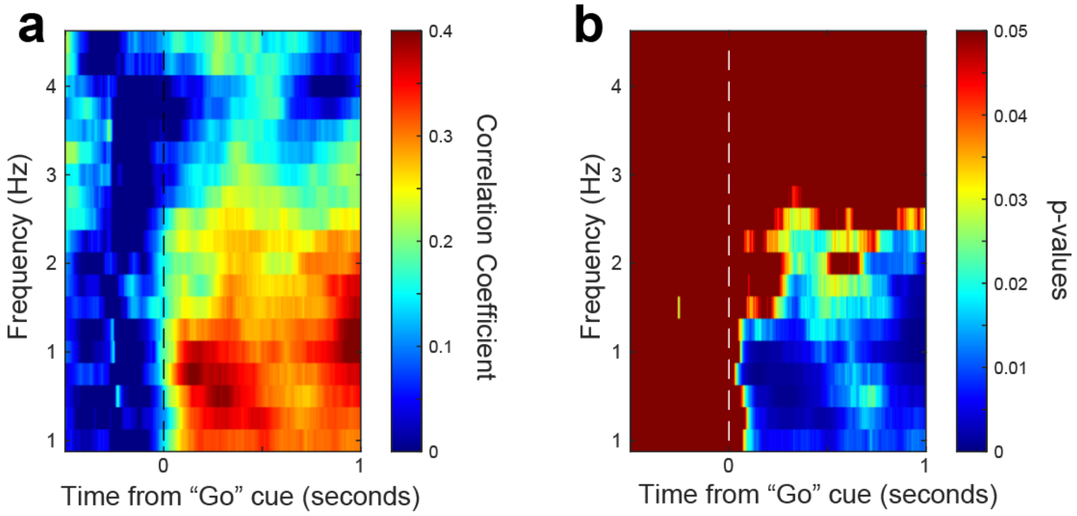

Supplementary Figure 11. Correlations of cue-triggered delta power with MOCA.

a). Correlation coefficient, and b) p-values for each point on time-frequency spectrogram from EEG electrode Cz. Data from 71 PD patients in Figure 2.

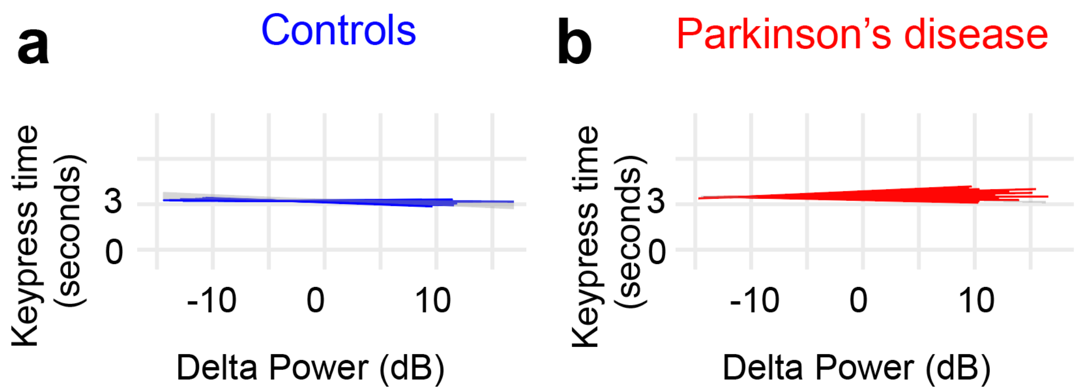

Supplementary Figure 12. Delta activity does not predict temporal variability for 3-second intervals. a-b) No clear relationship with delta power and time estimates was observed in controls or PD patients during 3-second interval timing task.

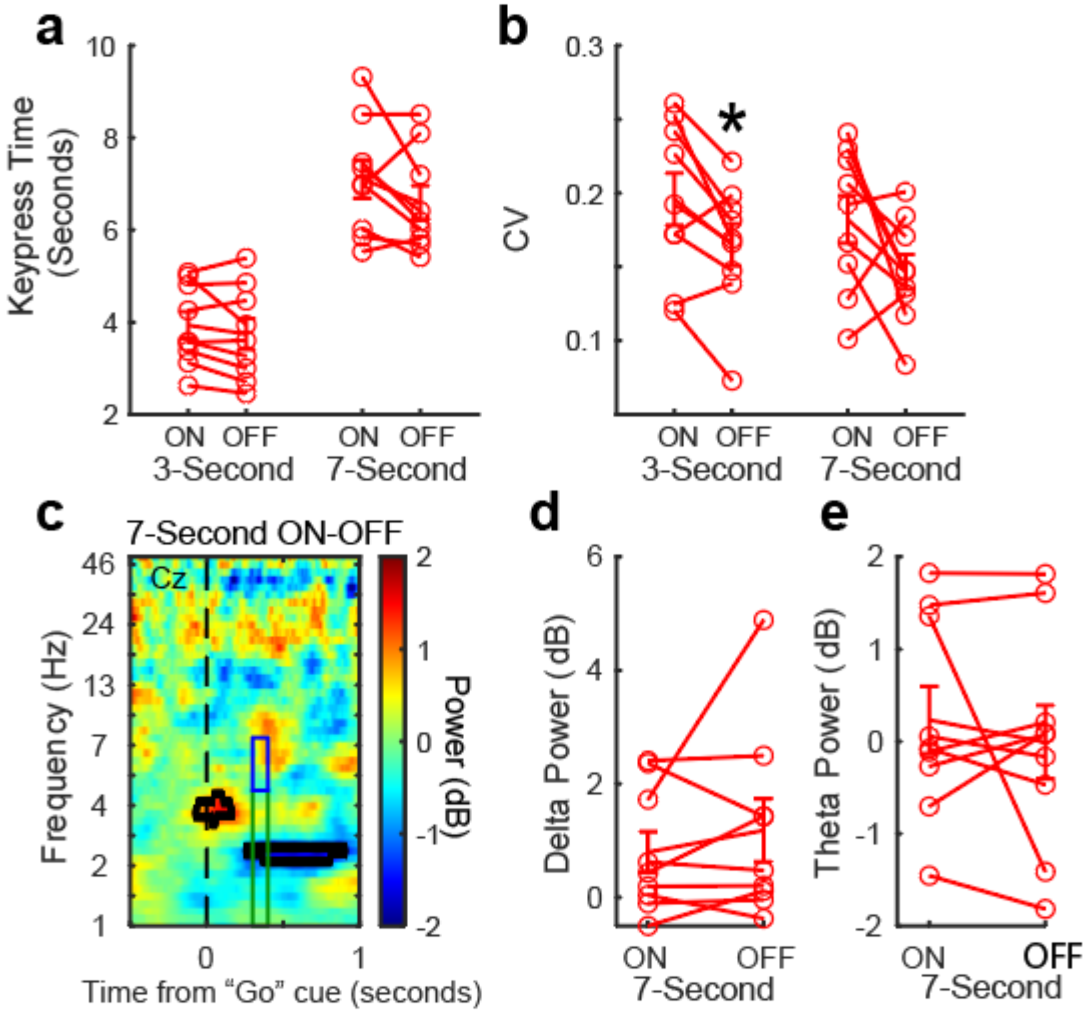

Supplementary Figure 13. Levodopa does not reliably affect midfrontal delta-theta activity. In PD patients, we examined a) mean response time, b) keypress CV, c) midfrontal cue-triggered activity from PD patients ON versus OFF levodopa. Areas outlined by solid black lines indicate $p<0.05$ via a t-test of activity in control compared to PD participants. d) Midfrontal cue-triggered delta activity (tf-ROI: green box) in c) and e) midfrontal cue-triggered theta-activity (tf-ROI: blue box in c) from PD patients ON and OFF levodopa. Data from 9 patients; *=$p<0.05$ via paired t-test.
